# Lonely in a crowd: investigating the association between overcrowding and loneliness using smartphone technologies

**DOI:** 10.1038/s41598-021-03398-2

**Published:** 2021-12-20

**Authors:** Ryan Hammoud, Stefania Tognin, Ioannis Bakolis, Daniela Ivanova, Naomi Fitzpatrick, Lucie Burgess, Michael Smythe, Johanna Gibbons, Neil Davidson, Andrea Mechelli

**Affiliations:** 1 Department of Psychosis Studies, Institute of Psychiatry, Psychology and Neuroscience, King’s College London, London, UK; 2 Outreach and Support in South London (OASIS) Service, South London and Maudsley NHS Foundation Trust, London, UK; 3 Health Services and Population Research Department, Centre for Implementation Science, Institute of Psychiatry, Psychology and Neuroscience, King’s College London, London, UK; 4 Department of Biostatistics and Health Informatics, Institute of Psychiatry, Psychology and Neuroscience, King’s College London, London, UK; 5 Nomad Projects, Sunbury Workshops, 24 Swanfield St., London, UK; 6 J & L Gibbons, 19 Swan Yard, London, UK

**Keywords:** Human behaviour, Lifestyle modification, Health policy, Epidemiology, Psychology and behaviour

## Abstract

Loneliness is a major public health concern with links to social and environmental factors. Previous studies have typically investigated loneliness as a stable emotional state using retrospective cross-sectional designs. Yet people experience different levels of loneliness throughout the day depending on their surrounding environment. In the present study, we investigated the associations between loneliness and social and environmental factors (i.e. overcrowding, population density, social inclusivity and contact with nature) in real-time. Ecological momentary assessment data was collected from participants using the Urban Mind smartphone application. Data from 756 participants who completed 16,602 assessments between April 2018 and March 2020 were used in order to investigate associations between momentary feeling of loneliness, the social environment (i.e. overcrowding, social inclusivity, population density) and the built environment (i.e. contact with nature) using multilevel modelling. Increased overcrowding and population density were associated with higher levels of loneliness; in contrast, social inclusivity and contact with nature were associated with lower levels of loneliness. These associations remained significant after adjusting for age, gender, ethnicity, education and occupation. The positive association between social inclusivity and lower levels of loneliness was more pronounced when participants were in contact with nature, indicating an interaction between the social and built environment on loneliness. The feeling of loneliness changes in relation to both social and environmental factors. Our findings have potential implications for public health strategies and interventions aimed at reducing the burden of loneliness on society. Specific measures, which would increase social inclusion and contact with nature while reducing overcrowding, should be implemented, especially in densely populated cities.

## Introduction

Despite the ever increasing levels of social connectivity, loneliness as a form of ‘social pain’ has become one of the defining issues of the modern society. Loneliness, defined as the ‘perceived sense of disconnection from others’, refers to the subjective emotional experience of not having one’s social need for relationships adequately met^1^. While initial research on loneliness focused on elderly populations^2^ or specific groups (e.g. childless, people with mental health issues)^3,4^, a recent study demonstrated that loneliness affects a much larger proportion of the society, with adults under 25 and above 65 feeling the loneliest^5^. Studies conducted in different countries tend to report similar loneliness rates with 1 in 10 adults feeling lonely^5–7^.

Loneliness can be a normative and adaptive experience, for instance in response to bereavement, however, it can also have profound detrimental effects on mental and physical health^3,8–10^. For example, the degree of loneliness predicts subsequent mental health symptoms, including depression, alcoholism, suicidal behaviour and cognitive decline leading to dementia, and physical health issues, including immune and cardiovascular disease^11,12^. It is therefore unsurprising that due to the reported incidence rates combined with these detrimental effects on health, loneliness is increasingly recognised as a major public health concern within the scientific community^4,8^ and amongst the general media^13^.

The term ‘loneliness’ is often associated with reduced social presence and social isolation^14^. However, mere social presence is not always linked with positive feelings, and loneliness and perceived social isolation have been found to be associated with, but distinct from, objective social isolation^8^. Perceived overcrowding tends to drive feelings of disconnectedness^15^. For example, Levine^16,17^ reported that when people are in highly populated areas they may feel that they do not have enough personal space, experience a higher level of social isolation and be less likely to help others in need. Chu et al.^18^ also showed how overcrowding might increase feelings of vulnerability, aggression and isolation. As the global population increases and more people move to large cities, with around 70% of the global population expected to live in urban areas by 2050^19^, it becomes critical to understand how overcrowding affects our experience of loneliness.

Related to overcrowding and at the other end of the disconnectedness-connectedness spectrum is the concept of social cohesion, which entails the interaction between the individual, the environment and the wider society^20^. A number of studies have implicated that social cohesion contributes to a sense of belonging within a community (i.e. social inclusion), which in turn promotes behaviours that have a positive effect on mental health, especially in time of distress^21,22^. Therefore, the feeling of social inclusivity within a particular sector of society might lessen the feeling of loneliness.

In addition to social aspects such as overcrowding and social inclusivity, the experience of loneliness is thought to be influenced by features of the built environment. In particular, studies have suggested that green and blue spaces provide opportunities for socialising outdoor which in turn increases the sense of community belonging and social cohesion^15,23–25^. Other studies have suggested that, while green and blue spaces might not necessarily facilitate the creation or maintenance of social contact per se, they might promote general psychological wellbeing including greater sense of trust, acceptance and belonging^26,27^. Indeed, further research into the ‘sense of place’ and ‘attachment to neighbourhood’ found that the sense of community can be affected by both physical (e.g. housing, traffic green spaces) and social (e.g. community size, type and density) aspects of the environment^28–30^.

Most of the previous studies have used population-based surveys or questionnaires to explore prevalence of loneliness (e.g. BBC’s Loneliness Experiment) or how loneliness affects aspects of mental or physical health^4^. While these studies have traditionally focussed on loneliness as a stable emotional state, recent studies have begun utilising advancing technologies to explore dynamic changes in loneliness as people go about their daily lives^31,32^. Although one might have a general feeling of loneliness, as with all emotional states, this is unlikely to be a static condition; in particular, a feeling of loneliness at a given moment may be influenced by changes in the surrounding social and built environment. In addition, while previous studies have investigated the link between loneliness and certain features of the environment (e.g. overcrowding), few of them have explored how multiple aspects of the built and social environment may interact with each other to modify the feeling of loneliness. Lastly, many previous studies have relied on cross-sectional population-based surveys which do not collect real-time information as people go about their daily lives, thus limiting ecological validity and largely overlooking dynamic changes over time^33^.

To assess the dynamic changes over time, the present study employs the Ecological Momentary Assessment (EMA) methodology to record individual experiences in ‘real-time’^34,35^. This makes EMA particularly well-suited to record the effects of the environment as it is experienced, reducing recall biases while increasing ecological validity. In particular we used the Urban Mind smartphone application (https://www.urbanmind.info;^34^) to investigate how social (i.e. perceived overcrowding and social inclusivity) and physical (i.e. contact with nature) aspects of the environment affect the feeling of loneliness in the moment. In addition, while most of the past studies focused on discrete populations, here we employed a large convenience sample of people in the general population (N = 2175) who have downloaded and used the Urban Mind app between April 2018 and March 2020.

Our primary research aim was to shed light on the associations between social and built environment and loneliness. We hypothesised that (i) perceived overcrowding will be positively associated with momentary feelings of loneliness; (ii) perceived social inclusivity will be negatively associated with momentary feelings of loneliness; and (iii) contact with nature will be negatively associated with momentary feeling of loneliness. To provide an objective comparison to these findings, we also hypothesised that (iv) increased population density will be positively associated with momentary feeling of loneliness. Finally, we explored possible interactions of contact with nature on the associations between loneliness and perceived overcrowding, social inclusivity and objective population density.

## Methods

The current study received institutional review board (IRB) approval from the King’s College London Psychiatry, Nursing and Midwifery Research Ethics Subcommittees (LRS-17/18-6905). All research was performed in accordance with relevant guidelines and regulations. All participants confirmed they had read the study information and privacy policy and provided informed consent.

### Urban Mind app

The present study was conducted using data collected from an adapted version of the Urban Mind app. Detailed description an earlier version of the Urban Mind tool can be found elsewhere^34^, but a brief summary of the adapted version used in the current study is provided here. The Urban Mind app is a smartphone-based ecological momentary assessment tool available for both Apple iPhone and Android devices. Participants were recruited globally, over a period of 24 months (April 2018–March 2020) using various social media platforms, the project-related website, and by word of mouth. Participation in the study was self-selected and anonymous. Once an individual downloaded and installed the app, they were presented with information about the study and were asked to provide informed consent. After consent was provided, participants were requested to complete a baseline assessment. This baseline assessment collected information regarding demographics (e.g. age, gender, ethnicity), socioeconomics (e.g. education, occupation), sleeping patterns (e.g. usual wake and sleep times) and self-reported mental health history (e.g. current and past mental health diagnoses).

Following the baseline assessment, the app scheduled a total of 42 ecological momentary assessments during the following 14 days (3 assessments per day). These assessments were scheduled based on the participants’ baseline-reported sleep schedule. The timeframes when the participants were awake were divided into 3 equal intervals, and an assessment was randomly scheduled within each window. Once an assessment was available, the app would prompt a participant to respond within 1 hour before the assessment was marked as incomplete. This allowed users to complete the assessment while minimising interruptions to their everyday activities. These momentary assessments collected information about an individual’s perceived built and social environment, and their location via GPS-based geotagging. After each assessment, participants were prompted to capture and submit a photograph of the ground and an 8-s audio clip of their surrounding environment. These images and audio clips were not included in the statistical analysis but were used to promote participant engagement and disseminate the project on our social media and website (www.urbanmind.info).

### Participants

During the 24-month recruitment period, 2175 participants downloaded the Urban Mind app and completed the baseline assessment. Of this sample, 756 participants completed at least 25% of the assessments (a minimum of 11 out of 42 assessments), 397 participants completed at least 50% of the assessments (21 out of 42 assessments), and 113 participants completed at least 75% of the assessments (32 out of the 42 assessments).

### Measures

#### Overcrowding

Perceived overcrowding derived based on a single item, “Does it feel overcrowded where you are right now?”, within each Urban Mind momentary assessment. Participants could respond with either “No”, “Not sure” or “Yes”. Perceived overcrowding was utilised as a binary variable within analyses, with “No” and “Not sure” combined into a category (0) and “Yes” as the other category (1).

#### Perceived social inclusivity

Momentary feelings of social inclusivity were derived from the sum of 3 questions during each Urban Mind momentary assessment. The items asked participants to think about the people in the neighbourhood at the time of the assessment: (1) “Do you feel welcome amongst them?” (2) “Do you feel they would be willing to help you?” and (3) “Do you feel they share the same values as you?”.

Participants responded with either “No” (1), “Not sure” (2) or “Yes” (3), and the scores were summed to create a “social inclusivity” score ranging between 3, indicating low perceived social inclusivity, and 9, indicating high perceived social inclusivity.

#### Contact with nature

Perceived contact with nature was derived from 5 items in the Urban Mind assessments: (1) “Can you see plants right now?” (2) “Can you see trees right now?” (3) “Can you see the sky right now?” (4) “Can you see or hear birds right now?” and (5) “Can you see water right now?”. Participants could respond to each of the questions with either “No”, “Not sure” or “Yes”. Contact with nature was included in our models as a binary variable, with “No contact” (0) if a participant answered either “No” or “Not sure” to all 5 nature questions, or “Contact with nature” (1) if a participant answered “Yes” to at least one nature question.

#### Population density

**Figure 1:**
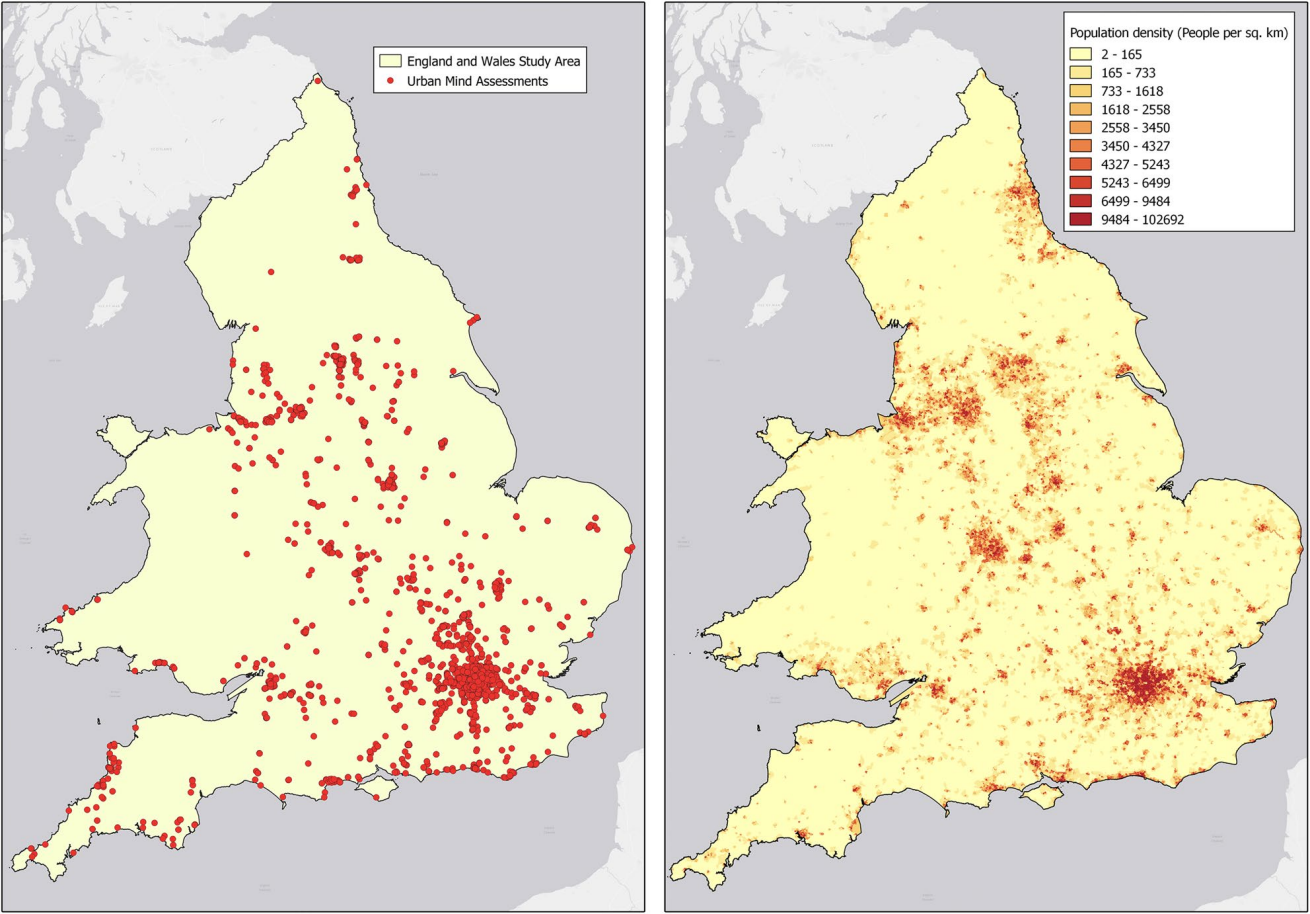
(Left) England and Wales sample Urban Mind assessment locations. (Right) Population density of LSOAs in England and Wales study area. The maps were created in QGIS 3.20.2 with images available at ArcGIS (https://www.arcgis.com/home/item.html?id=8a2cba3b0ebf4140b7c0dc5ee149549a), and the Office for National Statistics (https://data.gov.uk/dataset/11302ddc-65bc-4a8f-96a9-af5c456e442c/counties-and-unitary-authorities-december-2016-full-clipped-boundaries-in-england-and-wales; https://www.ons.gov.uk/peoplepopulationandcommunity/populationandmigration/populationestimates/datasets/lowersuperoutputareapopulationdensity).

Objective measures of population density of each Lower-layer Super Output Area (LSOA) within England and Wales were obtained from the Office for National Statistics^36^. These measures, population per total square area of the LSOA ($\frac{persons}{{km}^{2}}$), were estimates of usual resident population from mid-2018 and ranged from the least densely populated LSOA of 2 persons/km^2^ to the most densely populated LSOA of 102,692 persons/km^2^. A subsample of Urban Mind assessments completed within England and Wales were spatially linked to the corresponding LSOA and population density value for that LSOA. The population density values of the 1307 LSOAs included in our sample were divided into deciles for the current analysis (see Fig. 1 for approximate locations of Urban Mind assessments completed within England and Wales and an LSOA resolution population density heatmap). Time-varying population density exposures over 14 days of the sub-sample of participants in England and Wales who completed more than 75% of assessments are reported in Supplementary Fig. 1.

#### Momentary loneliness

Feelings of momentary loneliness were assessed using a single, 5-point Likert-scale item regarding an individual’s feelings during the momentary assessment: “Right now I am feeling lonely”. The participant would select a response ranging from “Strongly disagree” (1) to “Strongly agree” (5).

#### Covariates

To account for the effects of potential confounders, the statistical models were adjusted for covariates. This included participant demographic information collected during the initial baseline assessment, such as age, gender, ethnicity, education and occupation.

#### Statistical analysis

All statistical analyses were performed with STATA/SE 15. Longitudinal associations between momentary feelings of loneliness and self-reported perceived social inclusivity, overcrowding, contact with nature and objective population density were investigated using random intercept ordinal logistic regression models and expressed as odds ratios (OR) and 95% confidence intervals (CI). All models were adjusted for the following potential confounders: age, gender, ethnicity, education and occupational status of the participants.

#### The association between features of the social and built environment and momentary loneliness

The main statistical analysis focused on individuals who had completed at least 50% of the assessments (*n* = 397), with sensitivity analyses on different priori-defined samples involving participants who had completed at least 25% of the assessments (*n* = 756) and those who completed at least 75% of the assessments (*n* = 113). The analyses were run as univariate models unadjusted for confounders and again as multivariate models adjusted for confounding variables. In order to address missing data issues due to skipped assessments, all models were rerun using the STATA ice routine, an implementation of the Multiple Imputations with Chained Equations (MICE) procedure^37^. While this does not increase the tested sample sizes, this allowed us to impute the missing assessments from our samples. Our results using the MICE procedure were then compared with our results with the original analysis under the missing at random (MAR) assumption^38^, which assumes that the probability of missing assessments may differ between participants due to observed variables.

Interaction effects of contact with nature on the associations between (1) loneliness and perceived social inclusivity; (2) loneliness and overcrowding; (3) loneliness and objective population density, were assessed by including interaction terms in the models and are expressed as ratios of odds ratios (ROR) and 95% confidence intervals.

In order to investigate the relationship between momentary feelings of loneliness and objective measures of population density, a statistical analysis was conducted on an England and Wales subsample of 3,952 assessments from 138 participants with a 50% response rate. A further two additional sensitivity analyses were conducted including 6,145 assessments from 274 participants with a 25% response rate and 1,145 assessments from 32 participants with a 75% response rate.

## Results

### Sample characteristics

The demographic characteristics of the participants are presented in Table 1.

**Table 1:** Descriptive statistics of the main and sensitivity samples.

Assessment response rate	≥ 11 out of 42 assessments (≥ 25%)	≥ 21 out of 42 assessments (≥ 50%)	≥ 32 out of 42 assessments (≥ 75%)
	Number (%)	Number (%)	Number (%)
Number of participants	*n* = 756	*n* = 397	*n* = 113
**Gender**			
Female	526 (69.6%)	278 (70.0%)	79 (69.9%)
Male	225 (29.8%)	117 (29.5%)	34 (30.1%)
Other	5 (0.7%)	2 (0.5%)	0 (0.0%)
**Age**	Mean: 33.7 SD: 12.5	Mean: 34.8 SD: 12.9	Mean: 35.1 SD: 13.2
	Range: 16–80	Range: 16–73	Range: 16–73
**Ethnicity**			
African	14 (1.9%)	9 (2.3%)	2 (1.8%)
Caribbean	3 (0.4%)	2 (0.5%)	1 (0.9%)
Caucasian	484 (64.0%)	264 (66.5%)	76 (67.3%)
East Asian	61 (8.1%)	22 (5.54%)	10 (8.9%)
South Asian	55 (7.3%)	26 (6.6%)	7 (6.2%)
Indigenous	5 (0.7%)	3 (0.8%)	1 (0.9%)
Latino/Hispanic	28 (3.7%)	17 (4.3%)	1 (0.9%)
Middle Eastern	7 (0.9%)	3 (0.8%)	2 (1.8%)
Mixed	34 (4.5%)	19 (4.8%)	6 (5.3%)
Other	65 (8.6%)	32 (8.1%)	7 (6.2%)
**Occupation**			
Student	227 (30.0%)	112 (28.2%)	35 (31.0%)
Employed	407 (53.8%)	221 (55.7%)	58 (51.3%)
Self-employed	74 (9.8%)	35 (8.8%)	10 (8.9%)
Retired	20 (2.7%)	12 (3.0%)	5 (4.4%)
Unemployed	28 (3.7%)	17 (4.3%)	5 (4.4%)
**Education**			
Less than high school	13 (1.7%)	7 (1.8%)	0 (0.0%)
High school	88 (11.6%)	46 (11.6%)	12 (10.6%)
Apprenticeship	40 (5.3%)	25 (6.3%)	6 (5.3%)
University	615 (81.4%)	319 (80.4%)	95 (84.1%)

	Observations (%)	Observations (%)	Observations (%)
Number of momentary assessments	*n* = 16,602	n = 11,193	*n* = 3958
**Contact with nature**			
No	3773 (22.7%)	2553 (22.8%)	913 (23.1%)
Yes	12,829 (77.3%)	8640 (77.2%)	3045 (76.9%)
**Overcrowding**			
No	15,210 (91.6%)	10,279 (91.8%)	3666 (92.6%)
Yes	1392 (8.4%)	914 (8.2%)	292 (7.4%)
**Momentary lonely rating**	Mean: 1.87 SD: 1.09	Mean: 1.88 SD: 1.09	Mean: 1.83 SD: 1.09
1—Strongly disagree	8378 (50.5%)	5614 (50.1%)	2111 (53.4%)
2	4195 (25.3%)	2850 (25.5%)	943 (23.8%)
3	2274 (13.7%)	1510 (13.5%)	487 (12.3%)
4	1281 (7.7%)	926 (8.3%)	311 (7.9%)
5—Strongly agree	474 (2.9%)	293 (2.6%)	106 (2.7%)
Social inclusivity score	Mean: 7.38 SD: 1.66	Mean: 7.36 SD: 1.66	Mean: 7.37 SD: 1.62

Number (%) refers to the number and percentage of participants in each sample. Observations (%) refers to the number and percentage of completed ecological momentary assessment observations in each sample.

The main sample, highlighted in underline, comprised of participants who completed at least 50% of momentary assessments and two sensitivity samples comprised of those who completed at least 25% and 75% momentary assessments, respectively. The participants who completed at least 75% of the assessments were included in all three samples. The participants who completed at least 50% of the assessments were included in the main and 25% minimum sample.

Our main analysis investigating the relationship between momentary feelings of loneliness, perceived social inclusivity, overcrowding and contact with nature was based on 11,590 assessments from 397 participants who completed a minimum of 21 of the available 42 assessments (> 50% response rate). We also ran two additional sensitivity analyses, including 17,358 assessments from 756 participants with a 25% response rate and 4071 assessments from 113 participants with a 75% response rate.

Our main sample of 397 participants who completed at least 50% of the assessments consisted of 278 females, 117 males and 2 identified as other, with an average age of 34.8. While 56% of our sample indicated that they were employed, our sample also included 112 (28.2%) students. A majority of our sample identified as Caucasian (approximately 67%). The demographic characteristics remained fairly consistent across the assessment response rates.

To address the final hypothesis, exploring the association between population density on feeling of loneliness, a subsample, limited to individuals with assessments completed within England and Wales, was analysed in order to use consistent measures of population density. Excluding assessments containing incomplete geolocation data and those completed outside England and Wales, 274 participants completed at least 25% of the assessments, 138 participants completed at least 50% of the assessments and 32 participants completed 75% of the assessments. The main England and Wales subsample of 138 participants (50% completion rate) consisted of 100 (72.5%) females, 37 (26.8%) males and 1 (0.7%) other (Supplementary Table 1), with an average age of 35.5. While 54% of our sample indicated that they were employed, our sample also included 42 (30.4%) students. A majority of our sample identified as Caucasian (approximately 71%). The demographic characteristics remained fairly consistent across the main and sensitivity samples (which partially overlap) and are presented in Supplementary Table 1.

### The association between crowding, social inclusivity, contact with nature and momentary loneliness

**Figure 2:**
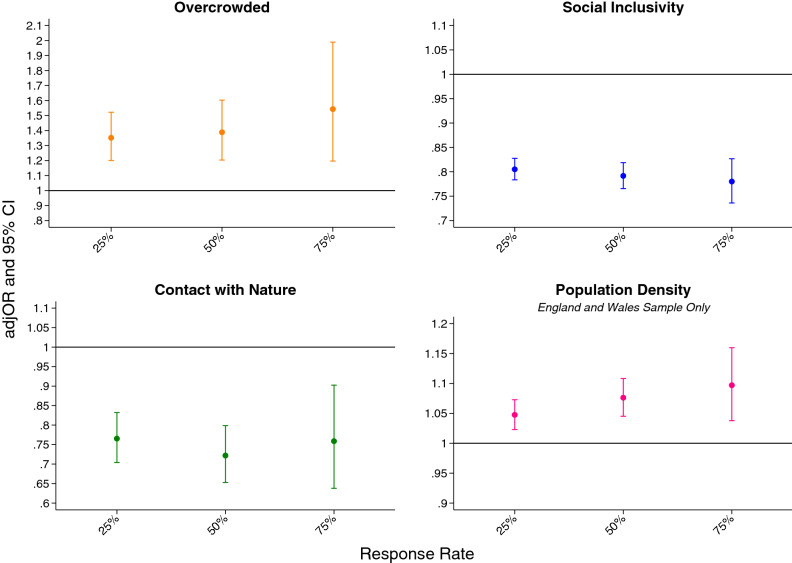
Adjusted odds ratios (adjOR) and 95% confidence intervals (CI) between self-reported momentary loneliness scores and feelings of social inclusivity, crowdedness, contact with nature and population density at the 25%, 50%, and 75% response rates.

Adjusting for age, gender, ethnicity, education and occupation, we observed a positive association between feelings of overcrowding and momentary loneliness (adjOR: 1.39; 95% CI: 1.20, 1.60). Conversely, social inclusivity was negatively associated with momentary loneliness (adjOR: 0.79; 95% CI: 0.77, 0.82). Contact with nature also appeared to be negatively associated with momentary loneliness (adjOR: 0.72; 95% CI: 0.65, 0.80) (see Fig. 2). These results were replicated when exploring univariate associations and when adjusting for age, gender, ethnicity, education and occupation across the three different samples (25%, 50% and 75% completion rates) (See Supplementary Table 2). These associations remained consistent when implementing the MICE procedure (See Supplementary Table 3).

### The association between population density and momentary loneliness

A positive association was found between the deciles of population density and momentary loneliness (*p* < 0.001) when adjusting for age, gender, ethnicity, education and occupation (Fig. 2). Specifically, the odds ratio between deciles of population density exposure and momentary loneliness was 1.08 (95% CI: 1.05, 1.11) after adjusting for age, gender, ethnicity, education and occupation. This association was also replicated as univariate associations and when adjusting for confounders across the different samples (25%, 50% and 75% completion rates) (Supplementary Table 4). Furthermore, this effect did not differ when implementing the MICE procedure to address missing data (Supplementary Table 5).

### Interaction effect of contact with nature on the association between loneliness and feelings of overcrowding, perceived social inclusion, and population density

In order to test if the above-mentioned associations of perceived overcrowding, social inclusivity, and objective population density on momentary feeling of loneliness were modified by contact with nature, we explored possible interactions between contact with nature and (1) perceived overcrowding; (2) social inclusivity; and (3) objective population density, on momentary feelings of loneliness. When adjusting for age, gender, ethnicity, education and occupation within our main sample, contact with nature appeared to interact with the association between perceived social inclusivity on momentary loneliness (*p* < 0.001) (Table 2—50% response rate, see model^b^). This ratio of odds ratios was 1.18 (95% CI: 1.11, 1.25), suggesting an augmenting effect of contact with nature on the association between perceived social inclusivity and loneliness. This interaction was also evident in additional statistical analyses exploring unadjusted associations and associations adjusted for confounders across the three sample sizes. These findings were replicated when implementing the MICE procedure. No other significant interactions were observed.

**Table 2:** Interaction ratio of odds ratios (ROR) of contact with nature on the associations between social inclusivity, perceived overcrowding, and population density and momentary loneliness.

	ROR (95% CI)	ROR (95% CI)	ROR (95% CI)	ROR (95% CI)	ROR (95% CI)
**Overall sample**	**25% response rate**^**a**^** (*n* = 756)**	**50% response rate**^**a**^** (*n* = 397)**	**75% response rate**^**a**^** (*n* = 113)**	**50% response rate**^**b**^** (*n* = 397)**	**50% response rate**^**c**^** (*n* = 397)**
Nature * Social Inc	1.13*** (1.07, 1.18)	1.18*** (1.11, 1.25)	1.20*** (1.08, 1.33)	1.18*** (1.11, 1.25)	1.09*** (1.04, 1.14)
Nature * Overcrowded	0.95 (0.74, 1.21)	0.90 (0.67, 1.21)	0.73 (0.42, 1.26)	0.90 (0.67, 1.21)	1.05 (0.79, 1.40)
**England and Wales sample**	**25% response rate**^**a**^** (*n* = 274)**	**50% response rate**^**a**^** (*n* = 138)**	**75% response rate**^**a**^** (*n* = 32)**	**50% response rate**^**b**^** (*n* = 138)**	**50% response rate**^**c**^** (*n* = 138)**
Nature * Population density	1.03 (0.98, 1.08)	1.02 (0.96, 1.08)	1.08 (0.97, 1.19)	1.02 (0.96, 1.08)	1.02 (0.96, 1.08)

Ratio of odds ratio (ROR) and 95% confidence intervals (CI) between self-reported momentary loneliness scores and (1) feelings of social inclusivity by contact with nature (2) crowdedness by contact with nature (3) population density by contact with nature at the 25%, 50%, and 75% response rates.

*p < 0.05.

**p < 0.01.

***p < 0.001.

^a^Unadjusted interactions between momentary loneliness scores and (1) feelings of social inclusivity by contact with nature; (2) crowdedness by contact with nature; (3) population density by contact with nature.

^b^Adjusted interactions controlled for age, gender, ethnicity, education and occupation.

^c^Adjusted interactions controlled for age, gender, ethnicity, education and occupation, plus the Multiple Imputations Chained Equations (MICE) procedure was employed for the main sample who completed more than 50% of the momentary assessments.

## Discussion

This the first study to examine aspects of the surrounding environment that might contribute to increase or decrease the feeling of loneliness as people go about their daily life. We hypothesised that loneliness would vary in response to social and physical aspects of the environment that a person experiences during the day. To assess the spatial and temporal variation of loneliness and the environment we used a smartphone app employing EMA.

We were consistent with our first hypothesis that perceived overcrowding would contribute to an increased feeling of loneliness. This hypothesis was confirmed, in line with past research on overcrowding which showed how people generally feel estranged and alienated in response to overcrowding and feel less able to connect with others, even when other are in distress^16,17^. This result is particularly important for urban planning, especially as the urban population is increasing as are high-rise residential buildings which are naturally prone to overcrowding^39,40^.

Our second hypothesis was that perceived social inclusivity would drive the opposite effect, of decreased feeling of loneliness. This hypothesis was also confirmed, with perceived social inclusivity linked with 21% (95% CI: 0.77, 0.82) reduced odds of heightened feelings of loneliness. This result is in line with previous studies^27,41^ which found that high perceived social capital and social cohesion are linked with decreased feeling of loneliness in the general population and in people who experienced a mental health crisis. In addition, the fact that we employed EMA allows us to make inferences beyond the participant’s own neighbourhood or primary place of living. Our study shows that, momentary location is also a crucial factor and when someone feels that people in the spatial vicinity are willing to help and share similar values, their feeling of loneliness decreases.

Our third hypothesis concerned the positive role of nature on feeling of loneliness. This hypothesis was also confirmed. Indeed, exposure to nature was linked with 28% reduced odds of loneliness. Importantly, the combined effect of exposure to nature and social inclusivity further reduced the feeling of loneliness. These results are line with a previous study that found that visiting green spaces is associated with higher social cohesion and in turn, with lower levels of loneliness^27^. A possible explanation for this is that natural space offers more opportunity to socialise. An alternative explanation is that natural elements contribute to an enhanced feeling of attachment to a place^28^ and hence might increase the perception of social inclusivity. On the other hand, the interaction between perceived overcrowding and nature was not significant, suggesting that the effect of nature on feeling of loneliness did not change depending on overcrowding. While this would seem to support the idea that environmental characteristics may be considered as separate constructs when assessing their influence on feelings of loneliness and mental wellbeing, we speculate that aspects of the built and social environment do not operate in isolation but are likely to interact with each other. Such interactive effects may be subtle and noisy, and therefore, it is possible that the interaction in question was not detected due to a lack of statistical power.

The Urban Mind app allows us to collect information on the perception of overcrowding, which can vary among individuals. However, the relationship between overcrowding and loneliness was supported by our additional analysis on the association between an objective measure of population density and loneliness that was carried out on an England and Wales sample (N = 138). Interestingly, within this sample, objective measures of population density were positively associated with subjective perception of overcrowding (see Supplementary Table 6), suggesting that people tended to feel more crowded when in higher populated areas. In our final hypothesis, we predicted that, similar to perceived overcrowding, population density would be associated with increased feeling of loneliness. This hypothesis was also confirmed. This result is important as it is supported by an objective measure that is readily available to policy makers and urban planners. This result is perhaps not surprising, as population density has been linked with other negative outcomes such as increased risk of depression, psychosis and anxiety^42,43^. However, population density can also translate into more access to services and more opportunities to socialise^39,44^. Our results might indicate that the “optimal” level of population density might have been surpassed. Loneliness is linked with adverse mental health and physical health outcomes and is becoming a public health problem^4^.

## Strengths and limitations

This study has a number of strengths. Firstly, we used a relatively large sample (N = 686, 25%) compared to some of the previous studies^27,41^. Moreover, the results are consistent across different priori-defined response thresholds. This is important as EMA is more ecologically valid compared to other research methods and is well suited to study variations of loneliness at different points in space and time.

Our perceived overcrowding results are also supported by the use of an objective measure of population density. In addition, participants did not stay in the same area: they travelled into objectively differently populated LSOAs and experienced different levels of loneliness (see Supplementary Fig. 1 for the time-varying population density exposures and loneliness scores over 14-days in a sub-sample of participants in England and Wales who completed more than 75% of the assessments). While overcrowding varies according to one’s perception, population density does not and it is an information immediately available to policy makers and urban planners.

This study has also a number of limitations. Firstly, the study employed a self-selected sample and hence might be prone to selection bias. The Urban Mind app is available to the general population and those who decide to download and use it might be people who have a specific interest in the effects of the environments in one’s wellbeing. While this is not a limitation per se, we might be missing out a proportion of the population who would not spontaneously engage with this topic. Despite this, as the smartphone app was available to the general population, the included participants were located in a variety of countries, suggesting that the findings are not confined to any single country or culture.

The second main limitation is related to the demographic characteristics of our sample. Our sample consisted of smartphone users with an average age of 34.8, a majority of which possessed a university-level education, and were employed or students and thus is not representative of the general population. This is particularly important since, according to previous studies, people under 25 and over 65 feel the loneliest^5,6^, and our data might not fully represent these age groups. Future studies would benefit from randomly recruiting from a more diverse sample including these groups.

A third limitation is missing data. As the app is available to the general population and there is no interaction with the research team during the EMA completion. As such, it is not possible to prompt completion and avoid missing data. Nonetheless, our results are consistent across different completion thresholds, including 25% (11 completed assessments over 42), and additionally, after implementing the MICE procedures to address missing data.

A fourth potential limitation concerns how loneliness and social inclusivity were assessed. We collected information about loneliness using a direct-single item within the EMA: “Right now I feel lonely”. Other studies have used indirect, multi-item questionnaires, such as the De Jong Gierveld Loneliness Scale^45^, to assess loneliness, with some finding gender and age differences between direct-single item compared with indirect multi-item questionnaires^7^. We collected information about social inclusivity using the sum of 3 questions which required participants to think about people in the neighbourhood at the time of the assessment: (1) “Do you feel welcome amongst them?” (2) “Do you feel they would be willing to help you?” and (3) “Do you feel they share the same values as you?”. Despite the importance of social inclusion as a global health outcome, there is currently a lack of a general consensus on how to best measure this^46^. For example, some studies have used multi-item questionnaires to assess several domains of social inclusivity^47,48^. In this study, the validity and reliability of the questions used to measure social inclusivity were not assessed. Therefore, we may not be able to generalise our findings to other definitions of social inclusion.

A fifth potential limitation is in regards to how information about contact with nature was collected. We asked participants whether they could see any natural features (e.g. can you see plants?), without considering whether they were actively interacting with them (e.g. gardening). Future studies may wish to collect additional information regarding the type of contact with nature.

Finally, the objective measure of population density was limited to England and Wales due to data availability and comparability across countries. We therefore cannot make inferences regarding the association between population density and loneliness to other countries.

Our results offer at least three key points for reflection around public health strategies:

1. The observation that loneliness is decreased by a feeling of social inclusivity provides indirect support to the idea of social prescribing—the indication to engage with social activities to improve health and wellbeing^49^. This might be particularly important for vulnerable populations, for example elderly people, those with reduced mobility and people with mental health issues^50^.
2. Loneliness shows spatial variation. This indicates that it is a dynamic rather than a static concept and that it can change in relation to the environment. In this sense it is not only important to focus on new residential areas but also to improve green space access in existing ones, for example by addressing existing inequalities^29^.
3. More research is required to explore what counteracts the adverse effects of increased population density. For example, how can we use natural spaces to enhance sense of community and decrease feeling of loneliness?

## Conclusions

The feeling of loneliness is increased by overcrowding and population density and decreased by perceived social inclusivity and contact with nature. The interaction effect of contact with nature on social inclusivity further decreases the feeling of loneliness.

In light of the well-established link between loneliness and physical and mental health, including mortality^10^, our findings have potential implications for public health strategies. Specifically, enhancing and preserving green spaces and improving walkability^15,51,52^ could help reduce the burden of loneliness in areas with high population density (e.g. high-rise buildings). Our findings also support the idea of “social prescribing”^49,50^, for example encouraging clinicians to suggest non-medical sources of support to promote health-seeking behaviours. Taken together, this and our prior findings^34^ highlight the importance for policy makers to consider measures which would increase social inclusion and contact with nature, while reducing overcrowding, in order to address feelings of loneliness in densely populated cities.

## Supplementary Information

Supplementary Information.
