# Supplementary material for: Lonely in a crowd: investigating the association between overcrowding and loneliness using smartphone technologies
Source: Sci Rep. 2021 Dec 20;11:24134. doi: 10.1038/s41598-021-03398-2 (PMC8688521; doi:10.1038/s41598-021-03398-2)
Supplement: Supplementary file 1 — Supplementary Information. [file 41598_2021_3398_MOESM1_ESM.docx]

**Supplementary Material**

**Supplementary Table 1.** Descriptive statistics of the three samples located in England and Wales at different completion rates.

| Assessment response rate | ≥ 11 out of 42 assessments (≥ 25%) | ≥ 21 out of 42 assessments (≥ 50%) | ≥ 32 out of 42 assessments (≥ 75%) |
| --- | --- | --- | --- |
|  | **Number (%)** | **Number (%)** | **Number (%)** |
| Number of participants | *n* = 274 (25%) | *n* = 138 (50%) | *n* = 32 (75%) |
| Gender | | | |
| Female | 199 (26.6%) | 100 (72.5%) | 27 (84.4%) |
| Male | 73 (72.6%) | 37 (26.8%) | 5 (15.6%) |
| Other | 2 (0.7%) | 1 (0.7%) | 0 (0.0%) |
| Age | Mean: 33.4 SD: 11.5 | Mean: 35.5 SD: 12.7 | Mean: 37.6 SD: 15.1 |
| Ethnicity | | | |
| African | 4 (1.5%) | 2 (1.5%) | 1 (3.1%) |
| Caribbean | 1 (0.4%) | 0 (0.0%) | 0 (0.0%) |
| Caucasian | 187 (68.3%) | 98 (71.0%) | 22 (68.8%) |
| East Asian | 15 (5.5%) | 9 (6.5%) | 3 (9.4%) |
| South Asian | 29 (10.6%) | 12 (8.7%) | 1 (3.1%) |
| Indigenous | 1 (0.4%) | 0 (0.0%) | 0 (0.0%) |
| Latino/Hispanic | 3 (1.1%) | 2 (1.5%) | 0 (0.0%) |
| Middle Eastern | 2 (0.7%) | 2 (1.5%) | 1 (3.1%) |
| Mixed | 10 (3.7%) | 4 (2.9%) | 1 (3.1%) |
| Other | 22 (8.0%) | 9 (6.5%) | 3 (9.4%) |
| Occupation | | | |
| Student | 91 (33.2%) | 42 (30.4%) | 13 (40.6%) |
| Employed | 140 (51.1%) | 75 (54.4%) | 15 (46.9%) |
| Self-employed | 29 (10.6%) | 12 (8.7%) | 3 (9.4%) |
| Retired | 4 (1.5%) | 2 (1.5%) | 1 (3.1%) |
| Unemployed | 10 (3.7%) | 7 (5.1%) | 0 (0.0%) |
| Education |  |  |  |
| Less than high school | 1 (0.4%) | 1 (0.7%) | 0 (0.0%) |
| High school | 25 (9.1%) | 16 (11.6%) | 2 (6.3%) |
| Apprenticeship | 14 (5.1%) | 6 (4.4%) | 2 (6.3%) |
| University | 234 (85.4%) | 115 (83.3%) | 28 (87.5%) |
|  | **Observations (%)** | **Observations (%)** | **Observations (%)** |
| Number of Momentary Assessments | *n* = 5902 | *n* = 3829 | *n* =1115 |
| Contact with Nature | | | |
| No | 1272 (21.6%) | 854 (22.3%) | 299 (26.8%) |
| Yes | 4630 (78.4%) | 2975 (77.7%) | 816 (73.2%) |
| Overcrowding |  |  |  |
| No | 5433 (92.1%) | 3523 (92.0%) | 1023 (91.8%) |
| Yes | 469 (7.9%) | 306 (8.0%) | 92 (8.2%) |
| Momentary Lonely Rating | Mean: 1.94 SD: 1.09 | Mean: 1.98 SD: 1.10 | Mean: 1.90 SD: 1.07 |
| 1 – Strongly disagree | 2756 (46.7%) | 1721 (45.0%) | 533 (47.8%) |
| 2 | 1578 (26.7%) | 1056 (27.6%) | 304 (27.3%) |
| 3 | 887 (15.0%) | 573 (15.0%) | 146 (13.1%) |
| 4 | 542 (9.2%) | 389 (10.2%) | 116 (10.4%) |
| 5 – Strongly agree | 139 (2.4%) | 90 (2.4%) | 16 (1.4%) |
| Social Inclusivity Score | Mean: 7.34 SD: 1.70 | Mean: 7.34 SD: 1.71 | Mean: 7.30 SD: 1.68 |
| Population Density Decile | Mean: 5.49 SD: 2.88 | Mean: 5.37 SD: 2.91 | Mean: 5.55 SD: 2.83 |
| *Number (%)* refers to the number and percentage of participants in each England and Wales sample. *Observations (%)* refers to the number and percentage of completed ecological momentary assessment observations in each sample.  The main sample comprised of participants who completed at least 50% of momentary assessments and two sensitivity samples comprised of those who completed at least 25% and 75% momentary assessments, respectively. The participants who completed at least 75% of the assessments were included in all three samples. The participants who completed at least 50% of the assessments were included in the main and 25% minimum sample. | | | |

**Supplementary Table 2.** Odds ratios (OR) and ratio of odds ratios (ROR) between self-reported momentary loneliness scores and exposures of interest.

|  | **25% response rate (*n* = 756)** | | **50% response rate (*n* = 397)** | | **75% response rate (*n* = 113)** | |
| --- | --- | --- | --- | --- | --- | --- |
|  | Unadjusted | Adjusted | Unadjusted | Adjusted | Unadjusted | Adjusted |
|  | **OR**  **(95% CI)** | **OR**  **(95% CI)** | **OR**  **(95% CI)** | **OR**  **(95% CI)** | **OR**  **(95% CI)** | **OR**  **(95% CI)** |
| Social Inclusivity | **0.80*****  **(0.78, 0.83)** | **0.81*****  **(0.78, 0.83)** | **0.79*****  **(0.77, 0.82)** | **0.79*****  **(0.77, 0.82)** | **0.78*****  **(0.74, 0.83)** | **0.78*****  **(0.74, 0.83)** |
| Overcrowded | **1.35*****  **(1.20, 1.52)** | **1.35*****  **(1.20, 1.52)** | **1.39*****  **(1.20, 1.60)** | **1.39*****  **(1.20, 1.60)** | **1.54****  **(1.20, 2.00)** | **1.54****  **(1.20, 1.99)** |
| Nature | **0.76*****  **(0.70, 0.83)** | **0.77*****  **(0.70, 0.83)** | **0.72*****  **(0.65, 0.80)** | **0.72*****  **(0.65, 0.80)** | **0.76****  **(0.64, 0.90)** | **0.76****  **(0.64, 0.90)** |
|  | **ROR**  **(95% CI)** | **ROR**  **(95% CI)** | **ROR**  **(95% CI)** | **ROR**  **(95% CI)** | **ROR**  **(95% CI)** | **ROR**  **(95% CI)** |
| Social Inc * Nature | **1.13*****  **(1.07, 1.18)** | **1.13*****  **(1.07, 1.18)** | **1.18*****  **(1.11, 1.25)** | **1.18*****  **(1.11, 1.25)** | **1.20*****  **(1.08, 1.33)** | **1.20*****  **(1.08, 1.33)** |
| Overcrowded * Nature | 0.95  (0.74, 1.21) | 0.94  (0.74, 1.20) | 0.90  (0.67, 1.21) | 0.90  (0.67, 1.21) | 0.73  (0.42, 1.26) | 0.73  (0.43, 1.26) |
| *Note: Odds ratio (OR) and 95% confidence intervals (CI) represent the odds of higher loneliness scores per category increase of exposure compared to the reference group. Interaction ratio of odds ratios (ROR) between self-reported momentary loneliness scores and 1) feelings of social inclusivity contact with nature; and 2) crowdedness by contact with nature at the 25%, 50%, and 75% response rates. Statistically significant associations (p* < 0.05*) are highlighted in bold.*  *Analyses were explored as crude associations and after adjusting for age, gender, ethnicity, education, and occupation.*  ** p* < 0.05  *** p* < 0.01  **** p* < 0.001 | | | | | | |

**Supplementary Table 3.** Odds ratios (OR) and ratio of odds ratios (ROR) between self-reported momentary loneliness scores and exposures of interest using the Multiple Imputation with Chains Equations (MICE) procedure.

|  | **25% response rate (*n* = 756)** | | **50% response rate (*n* = 397)** | | **75% response rate (*n* = 113)** | |
| --- | --- | --- | --- | --- | --- | --- |
|  | Unadjusted | Adjusted | Unadjusted | Adjusted | Unadjusted | Adjusted |
|  | **OR**  **(95% CI)** | **OR**  **(95% CI)** | **OR**  **(95% CI)** | **OR**  **(95% CI)** | **OR**  **(95% CI)** | **OR**  **(95% CI)** |
| Social Inclusivity | **0.78*****  **(0.76, 0.80)** | **0.78*****  **(0.76, 0.80)** | **0.78*****  **(0.76, 0.81)** | **0.78*****  **(0.76, 0.81)** | **0.78*****  **(0.74, 0.82)** | **0.78*****  **(0.74, 0.82)** |
| Overcrowded | **1.53*****  **(1.37, 1.71)** | **1.53*****  **(1.37, 1.71)** | **1.51****  **(1.24, 1.84)** | **1.51****  **(1.23, 1.83)** | **1.61*****  **(1.28, 2.03)** | **1.61*****  **(1.28, 2.03)** |
| Nature | **0.70*****  **(0.65, 0.74)** | **0.70*****  **(0.66, 0.75)** | **0.69*****  **(0.61, 0.78)** | **0.69*****  **(0.61, 0.78)** | **0.70****  **(0.59, 0.85)** | **0.71****  **(0.59, 0.85)** |
|  | **ROR**  **(95% CI)** | **ROR**  **(95% CI)** | **ROR**  **(95% CI)** | **ROR**  **(95% CI)** | **ROR**  **(95% CI)** | **ROR**  **(95% CI)** |
| Social Inc * Nature | 1.05  (1.00, 1.10) | 1.05  (1.00, 1.11) | **1.09*****  **(1.04, 1.14)** | **1.09*****  **(1.04, 1.14)** | **1.13***  **(1.03, 1.25)** | **1.13***  **(1.03, 1.25)** |
| Overcrowded * Nature | 1.00  (0.84, 1.19) | 1.00  (0.83, 1.19) | 1.05  (0.79, 1.40) | 1.05  (0.79, 1.40) | 0.87  (0.52, 1.45) | 0.87  (0.52, 1.45) |
| *Note: Odds ratio (OR) and 95% confidence intervals (CI) represent the odds of higher loneliness scores per category increase of exposure compared to the reference group. Interaction ratio of odds ratios (ROR) between self-reported momentary loneliness scores and 1) feelings of social inclusivity by contact with nature; and 2) crowdedness by contact with nature at the 25%, 50%, and 75% response rates.*  *All models employed the Multiple Imputation with Chained Equations (MICE) procedure. Analyses were explored as crude associations and after adjusting for age, gender, ethnicity, education, and occupation.*  ** p* < 0.05  *** p* < 0.01  **** p* < 0.001 | | | | | | |

**Supplementary Table 4.** England and Wales sample odds ratios (OR) and interaction ratio of odds ratios (ROR) between self-reported momentary loneliness scores and population density with exposures of interest.

|  | **25% response rate (*n* = 274)** | | **50% response rate (*n* = 138)** | | **75% response rate (*n* = 32)** | |
| --- | --- | --- | --- | --- | --- | --- |
|  | Unadjusted | Adjusted | Unadjusted | Adjusted | Unadjusted | Adjusted |
|  | **OR**  **(95% CI)** | **OR**  **(95% CI)** | **OR**  **(95% CI)** | **OR**  **(95% CI)** | **OR**  **(95% CI)** | **OR**  **(95% CI)** |
| Population Density Decile | **1.05*****  **(1.02, 1.07)** | **1.05*****  **(1.02, 1.07)** | **1.08*****  **(1.05, 1.11)** | **1.08*****  **(1.05, 1.11)** | **1.10****  **(1.04, 1.16)** | **1.10****  **(1.04, 1.16)** |
|  | **ROR**  **(95% CI)** | **ROR**  **(95% CI)** | **ROR**  **(95% CI)** | **ROR**  **(95% CI)** | **ROR**  **(95% CI)** | **ROR**  **(95% CI)** |
| Nature * Pop. Den Dec | 1.03  (0.98, 1.08) | 1.03  (0.98, 1.08) | 1.02  (0.96, 1.08) | 1.02  (0.96, 1.08) | 1.07  (0.97, 1.19) | 1.08  (0.97, 1.19) |
| *Note: England and Wales sample odds ratio (OR) and 95% confidence intervals (CI) represent the odds of higher loneliness scores per decile increase of population density compared to the reference group. Interaction ratio of odds ratios (ROR) between self-reported momentary loneliness scores and population density by contact with nature at the 25%, 50%, and 75% response rates within England and Wales.*  *Analyses were explored as crude associations and after adjusting for age, gender, ethnicity, education, and occupation.*  ** p* < 0.05  *** p* < 0.01  **** p* < 0.001 | | | | | | |

**Supplementary Table 5.** England and Wales sample odds ratios (OR) and ratio of odds ratios (ROR) between self-reported momentary loneliness scores and exposures of interest using the Multiple Imputation with Chains Equations (MICE) procedure.

|  | **25% response rate (*n* = 274)** | | **50% response rate (*n* = 138)** | | **75% response rate (*n* = 32)** | |
| --- | --- | --- | --- | --- | --- | --- |
|  | Unadjusted | Adjusted | Unadjusted | Adjusted | Unadjusted | Adjusted |
|  | **OR**  **(95% CI)** | **OR**  **(95% CI)** | **OR**  **(95% CI)** | **OR**  **(95% CI)** | **OR**  **(95% CI)** | **OR**  **(95% CI)** |
| Population Density Decile | **1.04*****  **(1.02, 1.07)** | **1.04****  **(1.02, 1.07)** | **1.08*****  **(1.05, 1.11)** | **1.07*****  **(1.04, 1.11)** | **1.10****  **(1.04, 1.16)** | **1.10****  **(1.04, 1.16)** |
|  | **ROR**  **(95% CI)** | **ROR**  **(95% CI)** | **ROR**  **(95% CI)** | **ROR**  **(95% CI)** | **ROR**  **(95% CI)** | **ROR**  **(95% CI)** |
| Nature * Pop. Den Dec | 1.03  (0.98, 1.08) | 1.03  (0.98, 1.08) | 1.02  (0.96, 1.08) | 1.02  (0.96, 1.08) | 1.08  (0.97, 1.20) | 1.08  (0.98, 1.20) |
| *Note: England and Wales sample odds ratio (OR) and 95% confidence intervals (CI) represent the odds of higher loneliness scores per decile increase of population density compared to the reference group. Interaction ratio of odds ratios (ROR) between self-reported momentary loneliness scores and population density by contact with nature at the 25%, 50%, and 75% response rates within England and Wales.*  *All models employed the Multiple Imputation with Chained Equations (MICE) procedure. Analyses were explored as crude associations and after adjusting for age, gender, ethnicity, education, and occupation.*  ** p* < 0.05  *** p* < 0.01  **** p* < 0.001 | | | | | | |

**Supplementary Table 6.** England and Wales sample odds ratios (OR) and ratio of odds ratios (ROR) between self-reported perception of overcrowding and objective population density.

|  | **25% Response Rate** | | **50% Response Rate** | | **75% Response Rate** | |
| --- | --- | --- | --- | --- | --- | --- |
|  | **Unadjusted** | **Adjusted** | **Unadjusted** | **Adjusted** | **Unadjusted** | **Adjusted** |
|  | **OR**  **(95% CI)** | **OR**  **(95% CI)** | **OR**  **(95% CI)** | **OR**  **(95% CI)** | **OR**  **(95% CI)** | **OR**  **(95% CI)** |
| **Population density and overcrowding** | **1.05****  **(1.01, 1.09)** | **1.05***  **(1.01, 1.09)** | **1.07****  **(1.02, 1.12)** | **1.06***  **(1.01, 1.12)** | **1.13****  **(1.03, 1.24)** | **1.14****  **(1.03, 1.25)** |
| *Note: Odds ratio (OR) and 95% confidence intervals (CI) represent the odds of higher overcrowding per increase of population density. Statistically significant associations (p* < 0.05*) are highlighted in bold.*  *Analyses were explored as crude associations and after adjusting for age, gender, ethnicity, education, and occupation.*  ** p* < 0.05  *** p* < 0.01  **** p* < 0.001 | | | | | | |

**Supplementary Figure 1.** Population density deciles and loneliness scores recorded by participants within England and Wales over 14 days of using the Urban Mind smartphone app.
